# Hereditary

**Published:** 1881-10

**Authors:** J. B. Chandler


### HEREDITARY.

J. B. CHANDLER.

The question of how far children are influenced for good or bad mentally by their ancestors, is just exciting attention which is likely to become practical under the influence of Prof. Darwin, as manifested by a letter from him, read before the Social Science Association, at Saratoga, some time since. The physical inheritance, the transmitted diseases, or peculiar bodily formations are too glaringly illustrated to be matters of inquiry, except so far as aiding in forming diagnoses for medical men, or in illustrating the theories of those who would seek to have our race profit by observations which have proven so useful in the development of high types in animal life. The present inquiry seems to have emanated from Mrs. Emily Talbot, and at her request Prof. Darwin gives his views in a short letter referred to above.

Of course, should the wish of the lady be carried out, and a careful noting of similarities manifested by children, in all the walks of life, toward the peculiarities and mental proclivities of their ancestors, and the work continued out through one or two generations, carefully noting the effect of education, and above all "circumstances" of which we are all said to be creatures, the result would be of inestimable use. The study is not an easy one, it will require a conscientious labor from parents, and with honesty of purpose must go a determinarion to withhold nothing good or bad of the children which may bear upon the case. Can such parents be found? It is doubtful, especially as the reward of their labor will be to others who shall enter upon life when the laborers have passed away

Whether the exhausting solution which science would demand shall be gained or not, why may not parents profit by the hints which such an inquiry gives them. Very few parents fail to note the proclivity in their family to some special disease handed down from the strain on one side or the other, and if possible they take measures as far as in their power to guard the peccant point. Why may not the same care be properly applied to mental proclivities which eminate from the same source? It will be seen that I assume that there is such a transmission.

It seems almost needless to direct attention to remarks constantly made, such as "She has a lovely disposition, so like her mother at her age," or "that boy is sharp as a steel trap, his father over again"—"a chip of the old block"—or "he is entirely unlike any of the family; I do not know where he gets it from," whilst some old maiden aunt will say, "you don't remember his grandfather or you would trace the resemblance." These are homely every-day remarks, but their very commonness adds to the claim I make that mental proclivities as well as physical ones are inherited.

Well! suppose they are; what is the use to which these inquiries can be applied; *cui bono?* If a child has transmitted to him unusual physical strength, would it not be advisable to train him so that his power would be properly applied, and not misapplied? If on the contrary his physical inheritance should be a bodily weakness that effectually debarred all hope of success in a walk of life requiring endurance and strength, the choice of a profession for him must be governed largely by that weakness, if you desire success.

The same rules apply with increased force to mental proclivities as inherited, and thus more deeply rooted than mere acquired habits. How shall they be detected? Who is to decide? Transmitted talents are not unfrequently manifested very late in life, and then, at their cropping out, never reach the perfection they would had the mine been struck at an early age.

Careful, conscientious study of the mental abilities and proclivities of their children is as much a duty of parents as the providing of food and raiment. The training of the first manifested tendril of mental strength or weakness so that it may ultimately do the least harm or develop the most good. Care should be taken to judiciously afford opportunity for the manifestation of a suspected inherited taste or ability, and if the suspicion should prove well founded, no other serious obstacle intervening, the child should be trained in that direction, and the result will undoubtedly be a success, because heart and hand will then work together.

I am aware that good and desirable proclivities may not always be inherited, but the bad qualities of ancestors may have control upon some unfortnnate. Should the progenitor have been a thief and the child manifest a decided kleptomaniac taste, then the bad is to be eradicated and the good cultivated. A skillful counterfeiter of coin might endow his child with a talent for imitating, which if honorably applied might tend to wealth and social position, but without proper home training the endowment might lead to the penitentiary.

These few homely illustrations I trust will call attention, at least, to the importance of the subject, the necessity for a careful study of the natural proclivities of children and the proper home influence to eradicate the bad and to cultivate and strengthen the good. No child with a mind, is so naturally bad, that he may not be redeemed with judicious training adapted especially to his peculiarities. No child so good but can be improved by a similar care or ruined by bad example.

There are other thoughts beyond these in this connection which are not likely to be of much practical use in the present state of social mind, but the time may, and probably will, come when cultivation shall have done so much for our race mentally that we shall be less slaves of animal propensities, and persons will not only avoid physical weakness in the selection of partners for life, but may also consult the mental proclivities to be strengthened or weakened by a union in which their children are to suffer or profit by the results, and their own personal reward will be largely in proportion to their success in this particular.

